# *O*-GlcNAcylation Is Involved in the Regulation of Stem Cell Markers Expression in Colon Cancer Cells

**DOI:** 10.3389/fendo.2019.00289

**Published:** 2019-05-08

**Authors:** Gabriela Fuentes-García, M. Cristina Castañeda-Patlán, Anne-Sophie Vercoutter-Edouart, Tony Lefebvre, Martha Robles-Flores

**Affiliations:** ^1^Departamento de Bioquímica, Facultad de Medicina, Universidad Nacional Autónoma de México, Mexico City, Mexico; ^2^Unité de Glycobiologie Structurale et Fonctionnelle, CNRS, UMR 8576, University of Lille, Lille, France

**Keywords:** colon cancer, *O*-GlcNAc, cancer stem cells, stemness, OGT, OGA

## Abstract

The dynamic *O*-linked-N-acetylglucosamine posttranslational modification of nucleocytoplasmic proteins has emerged as a key regulator of diverse cellular processes including several hallmarks of cancer. However, the role played by this modification in the establishment of CSC phenotype has been poorly studied so far and remains unclear. In this study we confirmed the previous reports showing that colon cancer cells exhibit higher *O*-GlcNAc basal levels than non-malignant cells, and investigated the role played by *O*-GlcNAcylation in the regulation of CSC phenotype. We found that the modification of *O*-GlcNAcylation levels by pharmacological inhibition of the *O*-GlcNAc-transferase enzyme that adds *O*-GlcNAc (OGT), but not of the enzyme that removes it (OGA), increased the expression of all stem cell markers tested in our colon malignant cell lines, and induced the appearance of a double positive (CD44+/CD133+) small stem cell-like subpopulation (which corresponded to 1–10%) that displayed very aggressive malignant phenotype such as increased clonogenicity and spheroid formation abilities in 3D culture. We reasoned that OGT inhibition would mimic in the tumor the presence of severe nutritional stress, and indeed, we demonstrated that nutritional stress reproduced in colon cancer cells the effects obtained with OGT inhibition. Thus, our data strongly suggests that stemness is regulated by HBP/*O*-GlcNAcylation nutrient sensing pathway, and that *O*-GlcNAc nutrient sensor represents an important survival mechanism in cancer cells under nutritional stressful conditions.

## Introduction

Colorectal cancer (CRC) is one of the most prevalent cancers and is a leading cause of cancer mortality worldwide. It is well-known that tumors are formed by different cells, and that among them, the cancer stem cell (CSC) subpopulation are proposed to be responsible for tumor initiation, drug and radiation resistance, invasive growth, metastasis, and tumor relapse (1). Several colorectal CSC markers have been reported to date, including CD133, CD44, CD24, CD166, and leucine-rich repeat-containing G-protein-coupled receptor 5 (LGR5) (1, 2). In addition, the CD44 isoform containing variant exon v6 (CD44v6) has been reported to play an important role in the progression, metastasis, and prognosis of CRC (3, 4).

*O*-linked β-N-acetylglucosamine (*O*-GlcNAc) protein modification has emerged as a critical regulator of diverse cellular processes, but its role in stem cells (SCs) and pluripotency has been poorly investigated so far and remains unclear. In this respect, several studies have suggested that O-GlcNAcylation promotes SC maintenance, and decrease in *O*-GlcNAcylation may be required for SC differentiation (5). Surprisingly, this highly dynamic modification of proteins is regulated only by two enzymes: the *O*-GlcNAc transferase (OGT), which adds the residue and the *O*-GlcNAcase (OGA), which removes it. Increased OGT activity has been shown to contribute to maintenance of stemness and to prevent differentiation to specific tissue types (6, 7). In addition, it has also been reported that increased OGT activity affects transcriptional activity of Sox2 and Oct4 SC marker proteins to maintain genomic stability, thereby maintaining self-renewal (5, 8).

Growth and proliferation of cancer cells tightly depend on their nutritional environment, particularly on glucose availability. It is well- known that SCs originating in hypoxic niches reprogram their metabolism from oxidative phosphorylation to aerobic glycolysis to increase glycolytic activity even in the presence of oxygen (Warburg effect). Glutamine is also taken up actively in embryonic SCs (9). However, even though the contribution of the metabolic and nutrient sensing pathways to stemness maintenance is recognized, there is very little understanding of the molecular mechanisms that link stemness to the nutrient-sensing pathways (7). However, among these, the hexosamine biosynthesis pathway (HBP) is probably most relevant. This pathway, which is triggered by increased glucose uptake, is helpful in biosensing glucose and routing it through a shunt pathway to make UDP-N-Acetyl glucosamine (UDP-GlcNAc) which is utilized for *O*- GlcNAc modification of cytosolic, nuclear and mitochondrial proteins (7). In this respect, it is well-known that *O*-GlcNAcylation adjusts protein function according to the nutritional status of the cell. Remarkably, increased glucose uptake has been demonstrated that leads to increased OGT activity (7, 8), and may be instrumental in regulating self-renewal not only in embryonic and hematopoietic SCs but also in CSCs. In this study we investigated the role played by *O*-GlcNAcylation in the establishment of CSC cell phenotype. Our data indicate that stemness is regulated by HBP/*O*-GlcNAcylation nutrient sensing pathway, and that *O*-GlcNAc nutrient sensor represents an important survival mechanism in cancer cells under nutritional stressful conditions.

## Materials and Methods

### Reagents and Antibodies

The following antibodies were used in the experiments: allophycocyanin (APC)-conjugated mouse anti-CD44 from BD Biosciences, phycoerythrin (PE)-conjugated mouse anti-CD133 from Miltenyi Biotec, rabbit anti-CD44, rabbit anti-CD133, and mouse anti-OGT from Abcam, Alexa 647-conjugated rabbit anti-mouse from Invitrogen; rabbit anti-β-tubulin from Cell Signaling Technology (Danvers, MA, USA); Alexa 488-conjugated goat anti-rabbit from Molecular Probes, Inc., (Eugene, OR, USA), mouse anti-*O*-GlcNAc (RL2) from Thermo Fisher Scientific; mouse anti-GAPDH from Santa Cruz Biotechnoloigy Inc (Sta. Cruz, CA, USA).

### Cell Lines

Primary SW480 and its derivative metastatic SW620 colorectal cell lines, express a truncated version of APC (Adenomatous polyposis coli), have constitutively active Wnt signaling and are prototype of KRAS-driven cancer cells (KRAS G12V, APC A1457T/K1462R, FGFR3 S400R, TP53 R273H, and STK11 G58S mutations) (10). These cancer cell lines and the non-malignant 112CoN colon cell line used here were purchased from American Type Culture Collection (ATCC; Manassas, VA, USA) and were authenticated in June 2017 by Short Tandem Repeat DNA profiling performed at the Instituto Nacional de Medicina Genómica (INMEGEN) in Mexico City.

### Cell Culture

112CoN cells were maintained in Dulbecco's Modified Eagle's medium (DMEM) supplemented with 10% (v/v) fetal bovine serum (FBS), antibiotics (120 mg/ml penicillin and 200 mg/ml streptomycin) and 2 mM L-glutamine. SW480 and SW620 cells were maintained in DMEM F-12 supplemented with 5% (v/v) FBS, antibiotics and 2mM glutamine. All cells were cultured in a humidified 5% (v/v) CO_2_ incubator at 37°C. For starvation cells were washed twice with phosphate-buffered saline (PBS, GIBCO/Invitrogen) and placed in HBSS buffer (GIBCO/Invitrogen).

### Western Blotting

Protein samples (30 μg) were separated by 10% SDS-PAGE followed by electrophoretic transfer onto nitrocellulose membranes (Bio-Rad, Hercules, CA, USA). The membranes were blocked with 5% (w/v) non-fat dry milk and incubated overnight at 4°C with the corresponding primary antibody. Detection was performed using the SuperSignal Kit (Pierce) with a horseradish peroxidase-conjugated second antibody. Actin or β-tubulin were used as control for equal loading.

### Immunofluorescence and Confocal Microscopy

Cells were seeded into 8-chamber culture slides (LabTek®) at 5 × 10^4^ cells/ml per chamber overnight. Then, samples were fixed with 1% (w/v) of paraformaldehyde in PBS for 10 min at room temperature (RT). Fixed cells were washed with PBS and cell permeabilization were performed with 1% (v/v) Triton X-100 in PBS for 5 min. Unspecific interaction sites were blocked with 3% (w/v) BSA/PBS. After washing, the slides were incubated with anti-*O*-GlcNAc, anti-CD133 or anti-CD44 primary antibodies (Abcam) diluted in 1% (m/v) BSA/PBS overnight at 4°C in darkness. Cells were washed with PBS, followed by incubation with secondary Alexa Fluor 647-conjugated goat anti-rabbit or Alexa 488-conjugated goat anti-mouse antibodies (Invitrogen) for 2 h at RT. Chambers were incubated with DAPI (SIGMA Aldrich) in PBS for 5 min at RT. After washing three times with PBS, the slides were mounted with Vectorshield® (Vector Labs, CA). Cells were examined under a Nikon A1R+ STORM confocal microscopy. Pictures were analyzed with ImageJ.

### Flow Cytometry

For membrane staining of CD44 and CD133, 1 × 10^4^ cells were detached with EDTA-PBS 10 mM solution, scraped gently and collected by centrifugation at 500 g. Pellet were washed twice with PBS and samples were incubated with anti-CD44-APC coupled (BD Bioscience), anti-CD133-PE-coupled (Myltenyi), or with a mix of CD44/CD133 during 20 min at 4°C in darkness. Then, PBS were added and cells were newly centrifuged. Finally, samples were analyzed with an Attune® cytometer.

For intracellular staining of OGT and *O*-GlcNAc, cells were detached in the same way but immediately they were fixed with 1% (m/v) of paraformaldehyde in PBS for 10 min at 4°C. Fixed cells were permeabilized with absolute methanol for 20 min on ice and unspecific interaction sites were blocked with 3% (m/v) BSA in PBS. Anti O-GlcNAc (RL2) or anti-OGT antibodies were added during 30 min at 4°C in darkness. Cells were washed one time and incubated with FITC-coupled anti-mouse or FITC-coupled anti-rabbit secondary antibodies. Cell were washed one time with PBS. Finally, samples were analyzed with a FACScalibur cytometer. All data were analyzed by FlowJo X software.

### Cell Sorting

As in flow cytometry, cells were detached in the same conditions. Samples were incubated with APC-conjugated anti CD44 (BD Bioscience), PE-conjugated anti-CD133 or a mix of ant-CD44/CD133 during 20 min at 4°C in darkness. Cells were washed. Samples were filtrated and collected in special cell cytometry sterile tubes. Cell sorting was performed to separate CD44+, CD133+ or CD44+/CD133+ (double positive) cells in a MoFlow Sorter. Later cells were seeded in different culture media types (DMEMF-12 with 5% (v/v) FBS, colony formation medium with ITS, or 3D culture media).

### Pharmacological Inhibitions

8 × 10^5^ cells were seeded in 6 -well culture plaques and incubated overnight. Then, for inhibition of OGT, cells were incubated 24 h in the absence (vehicle DMSO) or presence of Ac5SGlcNAc (50 μM final dilution), and for inhibition of OGA, were incubated 24 h in the absence (vehicle DMSO) or presence of Thiamet-G (1 μM final dilution). Cells were incubated at 37°C, and pictures of cell cultures for each treatment were taken at the end of incubation periods. The cells were then collected to perform flow cytometry and to make lysates for Western blotting.

### Apoptosis

Apoptosis was measured using the Annexin-V-FLUOS Staining Kit (Sigma-Aldrich) as recommended by the manufacturer's instructions. Briefly, after 24 h of incubation with Ac5sGlcNAc or ThiametG, cells were gently scraped (we did no use trypsin to detach cells to avoid the unspecific exposure of Annexin V), centrifuged and washed twice in PBS. Annexin V-FITC in the staining buffer and propidium iodide were added to cell suspension and incubated for 10 min at room temperature. Cell were washed and analyzed with Attune Flow Cytometer.

### Proliferation Assay

Proliferation was measured by labeling of cells with the fluorescent dye Carboxyfluorescein Diacetate Succinimidyl Ester (CFSE) to track generations of cells, since the associated fluorescence signal decreases by half with each cell division cycle. 5 × 10^4^ cells were incubated with CFSE (1 μM) in PBS during 20 min at 37°C. Then, cells were centrifuged and seeded on 12-well tissue culture plates. Cells were incubated in the absence or presence of Ac5sGlcNAc or ThiametG 24 h, then collected, centrifuged and analyzed by flow cytometry.

### Colony Formation Assay

After cell sorting, 500 cells were seeded in 6- well culture plaques with DMEM F-12 medium supplemented with insulin, transferrin and Selenite grow supplement (1X). After 2 weeks, pictures for each condition were taken and colonies on the plaque were counted.

### 3D Culture

After cell sorting, 500 cells were seeded in 6- well ultra-low attachment cell culture plaques with DMEM F-12 medium containing B27 (2% v/v) and EGF (20ng/ml) per duplicate. After 3 weeks, pictures for each condition were taken, and spheroids were collected to be lysed for Western blot analysis.

### Statistical Analysis

The data are expressed as the mean ± standard error of the mean (SEM). Statistical data analysis was performed using Student's *t*-test or a one-way-ANOVA with Tukey's multiple comparison test. A value of p < 0.05 was considered statistically significant.

## Results

### Expression Profile of Stem Cell Markers in Colon Cancer Cells Changes During Cancer Progression

**Figure 1:**
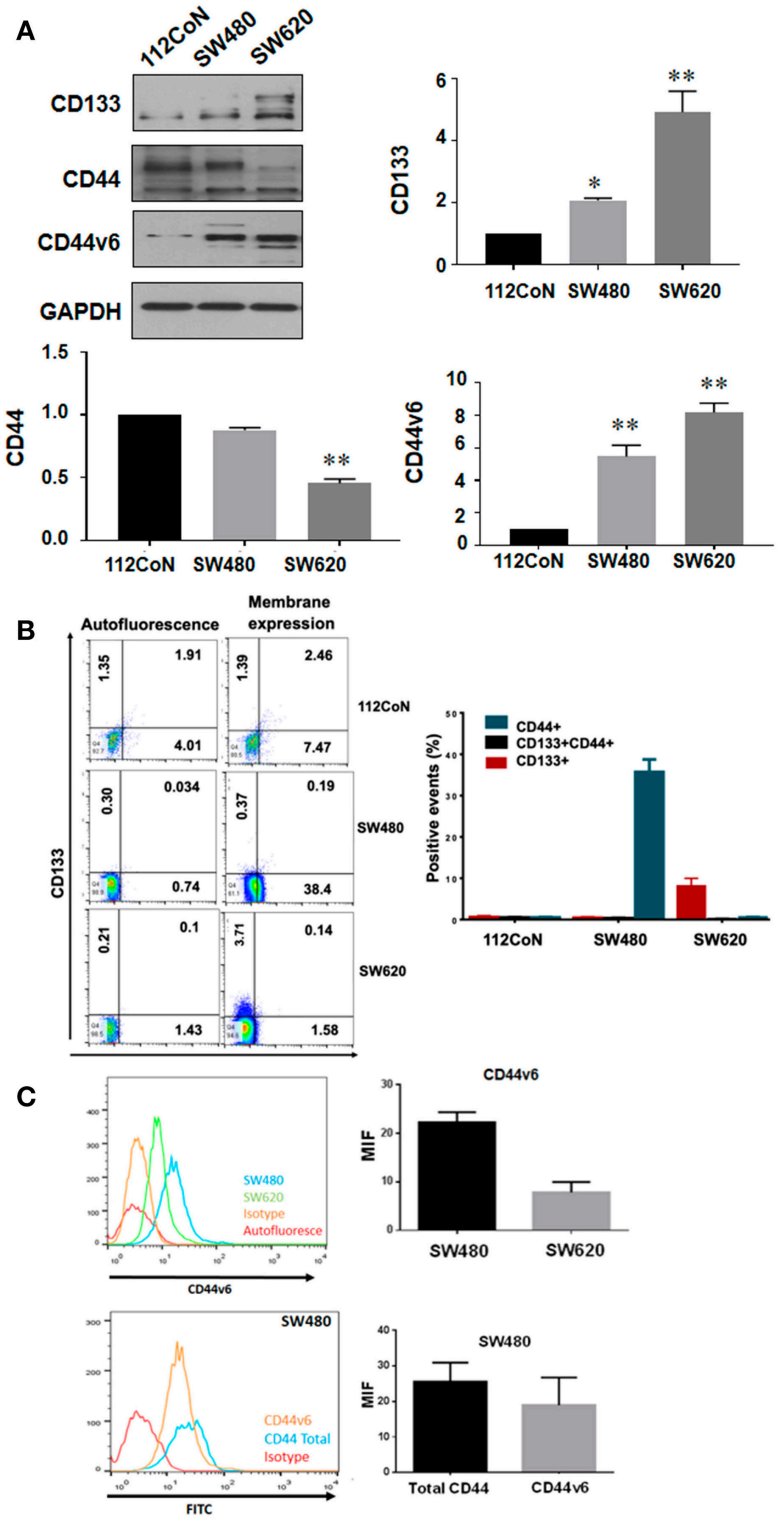
Expression of CD133, CD44, and CD44v6 in normal and colorectal cancer cell lines. **(A)** Western blot showing the expression of CD133, CD44, and CD44v6 on total lysates of the cell lines. GAPDH were used as a load control. The results shown are representative of at least three independent experiments using different cell preparations. A densitometric analysis of the expression levels found for each marker is shown at the right and the data represent the means ± SEM from at least three independent assays ^*^p = 0.01; ^**^p = 0.001. **(B)** Membrane expression of CD133+, CD44+, and CD133+CD44+ subpopulations in 112CoN, SW480, and SW620 cell lines. Acquisition of 1 × 10^4^ events. Anti-CD133 –PE-coupled and anti-CD44 –APC-coupled were used to stain proteins. MOFlow cytometer were used to acquired samples. **(C)** Membrane expression of CD44v6 in SW480 and SW620 cell lines. Acquisition of 1 × 10^4^ events. CD44v6 and a secondary antibody mouse anti-CD44v6 FITC-coupled were used to stain proteins. FACScalibur cytometer were used to acquired samples.

Several colorectal CSC markers have been reported to date, including CD133, CD44, CD24, CD166, and Lgr-5 (1, 2). CD44 isoform containing variant exon v6 (CD44v6) has also been reported to play an important role in the progression, metastasis, and prognosis of colorectal cancer (CRC) (3, 4). Because CD133 and CD44 have been widely validated as CSC markers in a variety of solid tumors including colon cancer, we examined their expression in human colon cancer cells. We selected as a model the primary SW480 colon carcinoma cell line and its derivative metastatic SW620 cell line which express a truncated version of APC, have constitutively active Wnt signaling and are prototype of KRAS-driven colon cancer cells in comparison with the non-malignant colon cell line 112CoN (Figure 1). Western blot analysis performed in total cells extracts shown in Figure 1A, indicated that CD133 marker, which appears as a doublet, is enriched in metastatic SW620 cells, while CD44, also seen as a doublet, was found mainly expressed both in non-malignant 112CoN cells and in primary cancer SW480 cells. The CD44 isoform, CD44v6, was found expressed only in both cancer SW480 and SW620 cells. Interestingly, when we analyzed the membrane expression of these stem cell markers by FACS (Figures 1B,C), we observed that neither CD133 nor CD44 are expressed in non-malignant colon cells at the cell membrane. In addition, we observed that SW480 cells only express CD44/CD44v6 but do not express CD133. Remarkably, SW620 cells, which are derived from a metastasis of the same tumor from which the SW480 cells were derived, only express CD133 and CD44v6, but do not longer express CD44. These results indicate therefore that there must have been a change in the expression profile of stem cell markers during malignant progression. Moreover, although non-malignant cells contain CD44, they do not express it at the cell membrane and in contrast, a great proportion of the total CD44 expressed at the cell membrane in SW480 cells corresponds to CD44v6, as shown in Figure 1C.

### Colon Cancer Cell Lines Have Increased *O*-GlcNAcylation Levels Compared With Non-malignant Colon Cells and Perturbation of These Levels Increased the Expression of Stem Cell Markers

**Figure 2:**
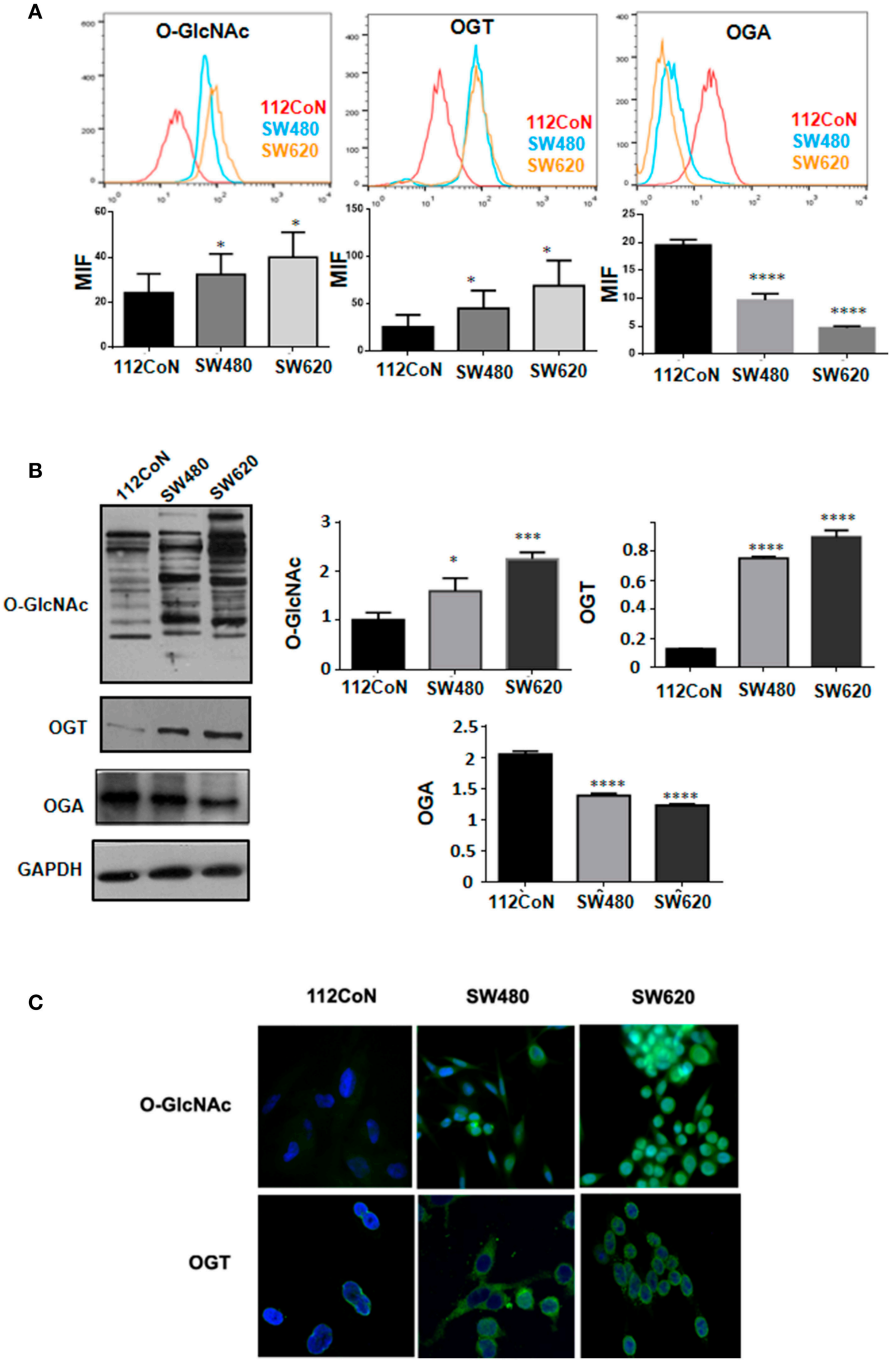
O-GlcNAcylation levels and OGT expression are higher in colon cancer cell lines compared with normal colon cell line while OGA expression diminished in colon malignant cells compared with non-malignant ones. **(A)** Flow cytometry. The data represent the means ± SEM from at least three independent assays ^*^p = 0.01; ^***^p = 0.001; ^****^p = 0.0001 compared with control 112CoN cells. **(B)** Western blot and **(C)** Immunofluoresce assay showing the different levels of O-GlcNAcylation and OGT expression in each cell line. Normal cells: 112CoN, cancer cells: SW480 and SW620.

Increased *O*-GlcNAcylation levels have been reported in diverse types of cancers including colon cancer (10, 11). To determine the levels of *O*-GlcNAcylation, OGT expression, and OGA expression in colon cancer cells compared to non-malignant colon cells, we performed FACS analysis, Western blot analysis, and immunofluorescence assays. The results shown in Figure 2A (FACS analysis), Figure 2B (Western blot analysis), and Figure 2C (immunofluorescence assays) clearly indicated that, as previously reported, the *O*-GlcNAcylation levels are higher in colon cancer cells compared to non-malignant cells. Consistent with this, Figures 2A,B show how the expression of OGT, which adds *O*-GlcNAc, appears increased while that of OGA, which removes it, appears diminished in colon malignant cells, compared with the expression found in colon non-malignant cells.

**Figure 3:**
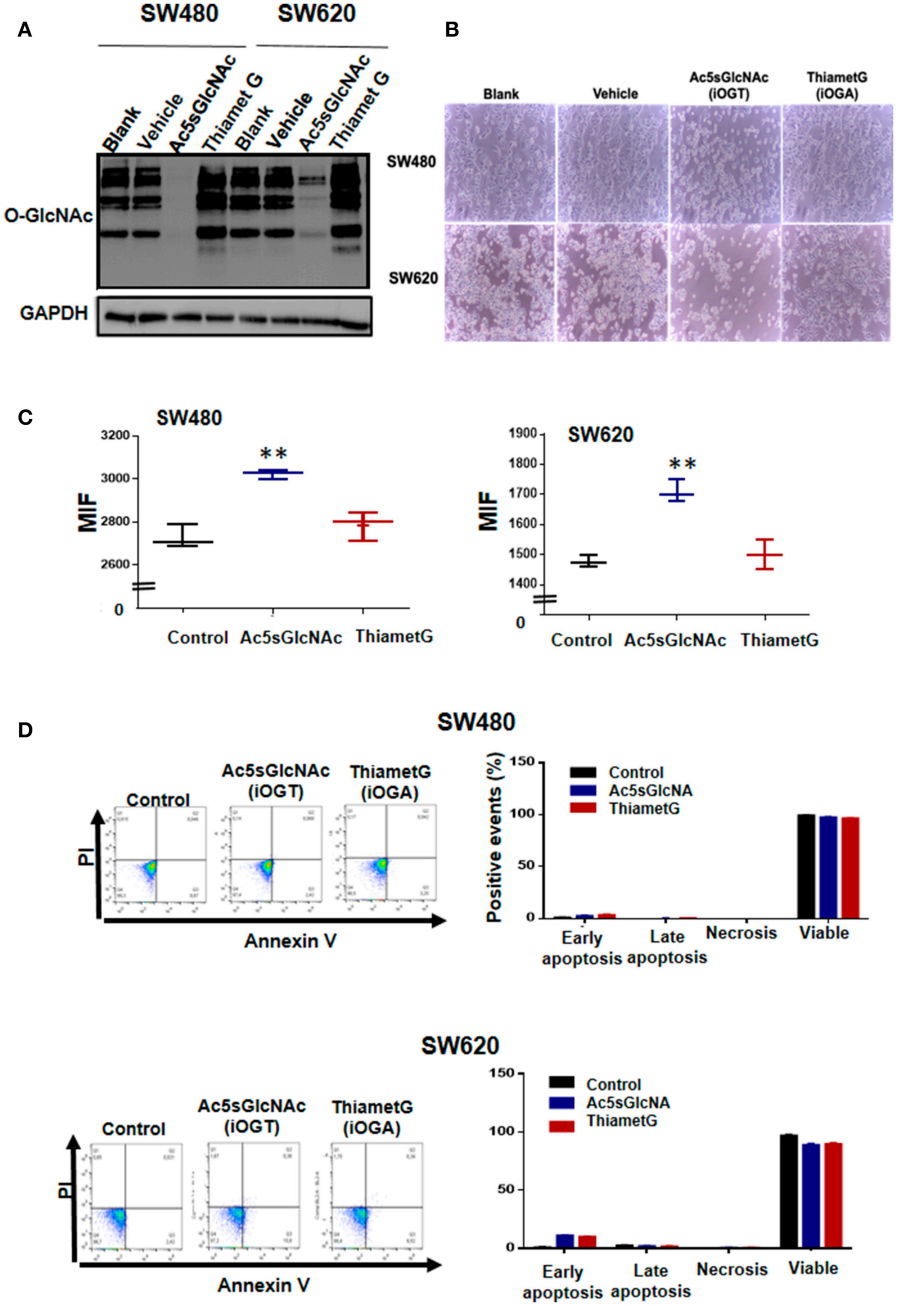
Effects of the inhibition of OGT and OGA on the proliferation and apoptosis rates of SW480 and SW620 cells. Cells were incubated during 24 h in the absence (vehicle) or presence of 50 μM Ac5sGlcNAc to decrease the levels of O-GlcNAc, or in the absence (vehicle) or presence of 1 μM Thiamet- G to inhibit OGA and increase them **(A)** Western blotting showing the effectiveness of the inhibitors on GlcNAc cellular levels. **(B)** After 24 h, pictures of each treatment were taken with a light microscopy, augmentation 40X. **(C)** Proliferation assays were performed by flow cytometry with CellTrace CFSE. Data represent the means ± SEM from at least three independent assays ^**^p = 0.001 (*t*-test) compared with control 112CoN cells. **(D)** Cell death was evaluated with the Annexin V-FITC Apoptosis Detection Kit. Dotplots show early, late, necrosis or viable cells. The data represent the means ± SEM from at least three independent assays.

We next investigated the effects produced by perturbation of *O*-GlcNAc levels on the expression of stem cell markers CD44 and CD133 by pharmacological inhibition of OGT or OGA in colon cancer cells. We first examined by Western blot the effectiveness of the OGT inhibitor Ac5SGlcNAc (50 μM) to decrease *O*-GlcNAc levels on cells and of the OGA inhibitor Thiamet-G (1 μM) to increase *O*-GlcNAc levels (Figure 3). As it can be observed in Figure 3A, as expected, when OGA was inhibited, a global elevation of protein *O*-GlcNAcylation was observed in both cancer cell lines, whereas inhibition of OGT induced a strong decrease in *O*-GlcNAc levels compared with controls in both cell lines. Pictures taken 24 h after incubation of cells with the OGT or OGA inhibitors are shown in Figure 3B. It is interesting to observe in this figure that in both cell lines the inhibition of OGT, but not of OGA, produced a decrease in the total number of cells seen in the pictures. Because the decreased amount of cells could be caused by a decrease in the proliferation rate or by an increase in the apoptotic cell death, we decided to analyze the impact of the inhibition of OGT or OGA on both processes in malignant cells. The results presented in Figure 3C indicate that the OGT inhibition negatively affected the proliferation of both SW480 and SW620 malignant cells, as reported before in other cancer cell types (10) and in colon cancer cells (12). The inhibition is visualized in the figure as a retention of the fluorescent compound CFSE in cells treated with Ac5sGlcNAc because they did not proliferate, while in control or in Thiamet-G - treated cells the fluorescence signal decreased with each cell division cycle. In addition, neither the treatment of cells with the OGT inhibitor nor with the OGA inhibitor significantly affected the apoptosis rate of both SW480 or SW620 cells, as shown in Figure 3D.

**Figure 4:**
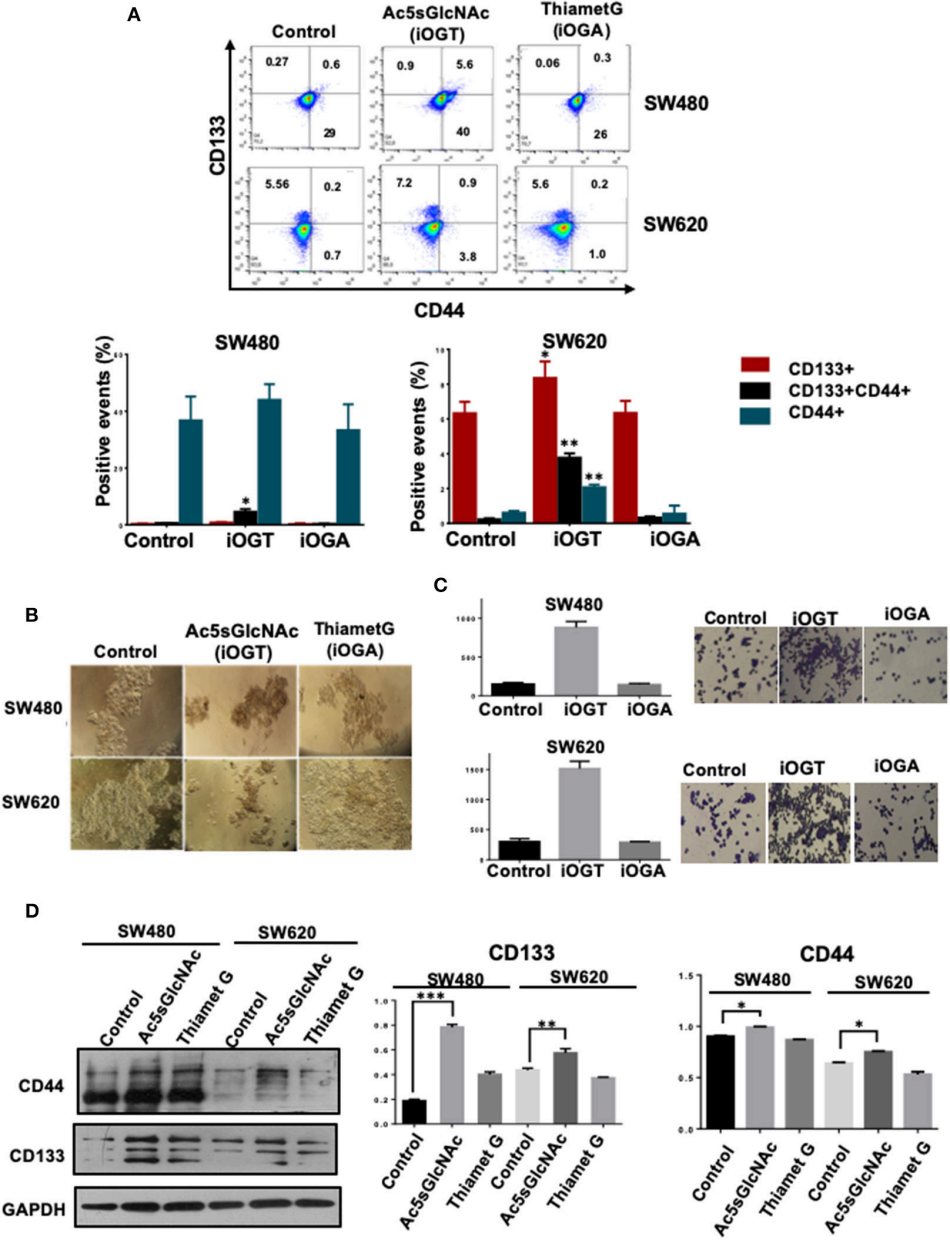
Inhibition of OGT promoted changes in the expression of CD44+ CD133+ cancer stem cell markers. Cell were incubated 24 h in the absence (DMSO as vehicle) or presence of 50 M Ac5sGlcNAc to decrease the levels of O-GlcNAc, or in the absence (vehicle DMSO) or presence of 1 M Thiamet G to inhibit OGA and increase them. **(A)** After 24 h of inhibition, expression of CD44+ CD133+ and CD44+CD133+ subpopulations was evaluated by flow cytometry. The data represent the means ± SEM from at least three independent assays ^*^p = 0.01; ^**^p = (*t*-test) compared with control. **(B)** Cells Spheroid culture. Total cell populations were cultured in ultra-low adherence six-well plates with medium supplemented with EGF and B27. After 2 weeks of incubation pictures of the spheres were taken. **(C)** Clonogenicity assay. Cells were cultured in DMEM F12 with ITS in the absence or presence of the OGT or OGA inhibitors to evaluate colony formation. Bar graphs shown represent the mean ± SEM of three independent experiments. **(D)** Western blot showing the expression of CD133 and CD44 on total cell lysates of the spheroid culture. GAPDH were used as a load control. The results shown are representative of at least three independent experiments using different cell preparations. A densitometric analysis of the expression levels found for each marker is shown at the right and the data represent the means ± SEM from at least three independent experiments. ^*^p = 0.01; ^**^p = 0.005; ^***^p = 0.001 (*t*-test) compared with control 112CoN non-malignant cells.

We next investigated the effects of the modification of *O*-GlcNAc levels on the expression of cancer stem cell markers by FACS analysis. The results presented in Figure 4A clearly indicated that the inhibition of OGA did not affect the expression profile of CD44 and CD133 stem cell markers in either SW480 cells or SW620 cells compared with control untreated cells. However, and in clear contrast, the inhibition of OGT in the metastatic SW620 cell line, induced the expression of CD44 and an increase in CD133 expression. In addition, and remarkably, OGT inhibition induced the appearance of a double positive CD44+/CD133+ cell subpopulation in both primary SW480 and metastatic SW620 cancer cell lines. Thus, these results suggested that the inhibition of OGT increases the stemness in colon cancer cells. Consistent with this, when we incubated both control or treated cells with the OGT or the OGA inhibitors in sphere formation and in clonogenic activity assays, only the Ac5sGlcNAc-treated SW480 or SW620 cells formed well-defined and condensed spheres as observed in Figure 4B, and the clonogenic activity only increased in the OGT-inhibited cells, as it can be seen in Figure 4C. Moreover, the analysis of the expression of CD44 and CD133 stem cell markers by Western blotting in both SW480 and SW620 control or treated cells showed a significant increase in the CD44 and CD133 expression only in the OGT-inhibited cells compared with the OGA-inhibited and with control cells (Figure 4D).

### Double Positive CD133/CD44 Stem Subpopulations Induced as Result of OGT Inhibition Display More Aggressive Phenotype Compared With Single Positive Subpopulations

**Figure 5:**
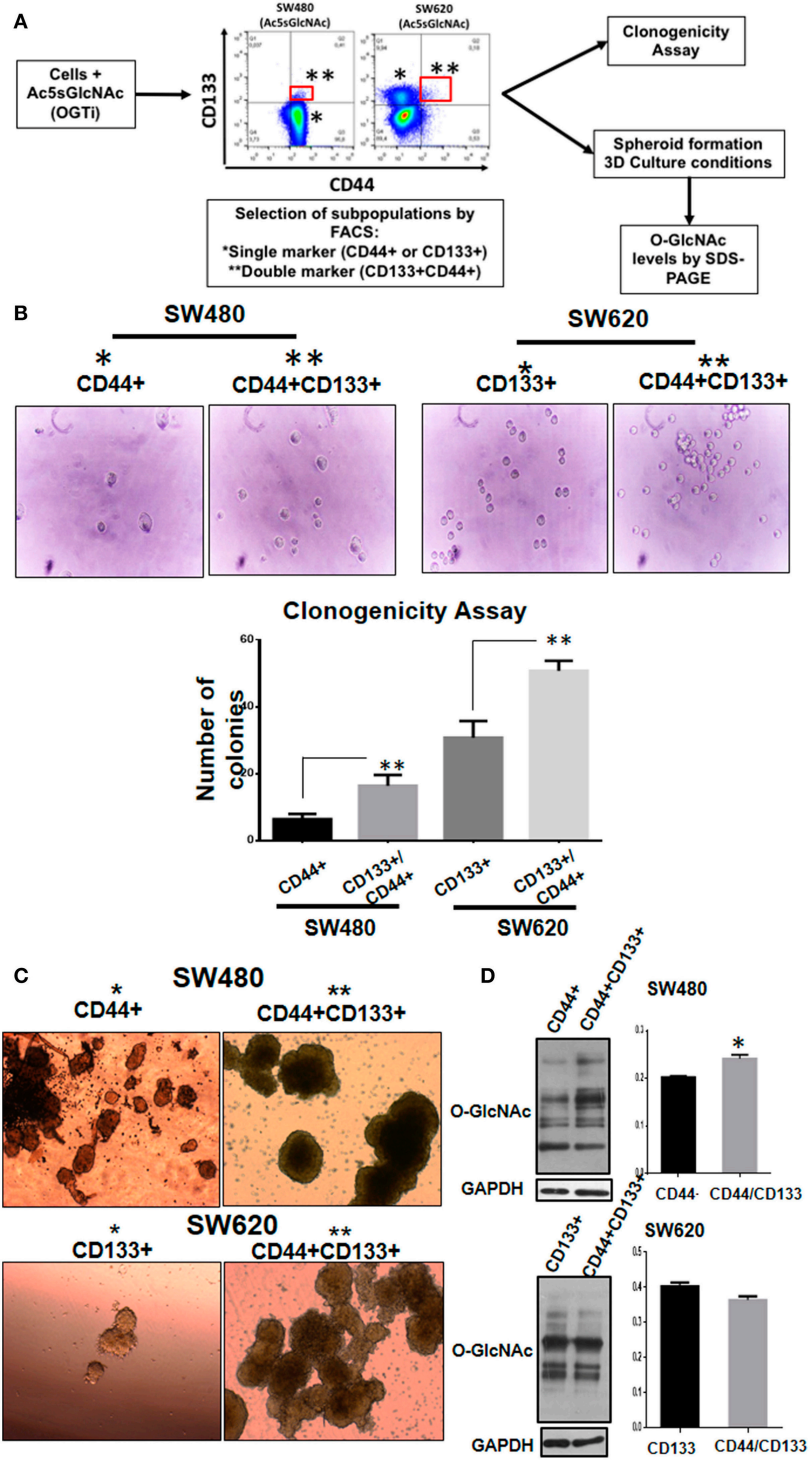
Double positive CD133/CD44 stem subpopulations induced as result of OGT inhibition display more aggressive phenotype compared with single positive subpopulations. **(A)** Schematization of the experimental approach used. **(B)** Clonogenicity assay. Isolation by FACS of the single and double positive populations was performed. Cells co-expressing CD44 and CD133 (marked in red) were isolated and grown under different culture conditions. Cells were cultured in DMEM F12 with ITS to evaluate colony formation. Bar graphs shown represent the mean ±SEM of three independent experiments. ^*^p = 0.01; ^**^p = 0.001 (*t*-test) compared with control. **(C)** Spheroid culture. Cells were cultured in ultra- low adherence 24-well plates with medium supplemented with EGF and B27. After 3 weeks of incubation pictures of the spheres were taken. For comparison, cells expressing only one marker (CD44 for SW480 and CD133 for SW620) were cultured under the same conditions. The spheres were lysed and levels of O-GlcNAc expression were evaluated. Isolation of cells by FACS -cell sorting was performed in MOFlow cytometer. **(D)** The spheres were lysed and levels of O-GlcNAc expression were evaluated by Western blotting. GAPDH were used as a load control. The results shown are representative of at least three independent experiments using different cell preparations. A densitometric analysis of the expression levels found is shown at the right and the data represent the means ± SEM from at least three independent experiments. ^*^p = 0.01 (*t*-test) compared with single positive CD44+ spheroids.

The appearance of double positive CD133/CD44 cancer cells has been characteristically found in several highly metastatic tumors of colon, liver, pancreas, and gallbladder (13–19). Therefore, we decided to investigate if this event correlated with a change to a more aggressive malignant phenotype. To this end, we analyzed the typical cancer stem cell traits such as clonogenic and spheroid formation abilities in the double positive CD44/CD133 cell subpopulations compared with the single positive subpopulations obtained as result of OGT inhibition. As depicted in Figure 5A, the stem cell subpopulations found in each colon cell line after incubation with the OGT inhibitor were isolated by FACS-cell sorting and cultured for analysis of clonogenicity and of their ability to form spheroids in 3D cultures. The results presented in Figure 5B show that as expected, SW620 cells, which are metastatic, clearly formed more colonies than primary SW480 cells. But interestingly, when single positive stem marker subpopulation was compared with double positive CD44/CD133 subpopulation in each cell line, the double positive displayed an increased clonogenicity ability, indicative of a more aggressive malignant phenotype (Figure 5B). We also perfomed 3D culture in selective media to induce colonosphere formation, and as shown in Figure 5C, we observed that both subpopulation types in either SW480 or SW620 cells had the ability to form spheres in the selective medium. However, the double positive stem cell subpopulation in either SW480 or SW620 cells formed much bigger and condensed spheroids than single positive marker subpopulations, particularly in the metastatic cell line SW620. Finally, the evaluation of the levels of *O*-GlcNAcylation in the isolated stem cell subpopulations derived from SW480 or SW620 cells treated with OGT inhibitor (Figure 5D) showed that whereas SW480 double positive stem cell subpopulation displayed higher levels of *O*-GlcNAc compared with their single marker counterparts, there was no significant change in the O-GlcNAc levels found in SW620 double positive stem cells compared with single positive stem cells. It must be taken into account that after exposure of stressful conditions, and once cells adapt to the growth conditions, the O-GlcNAc levels are recuperated. However, it is interesting to note that.

### Nutritional Stress Mimics OGT Inhibition Effects in Cancer Stem Cell Expression

**Figure 6:**
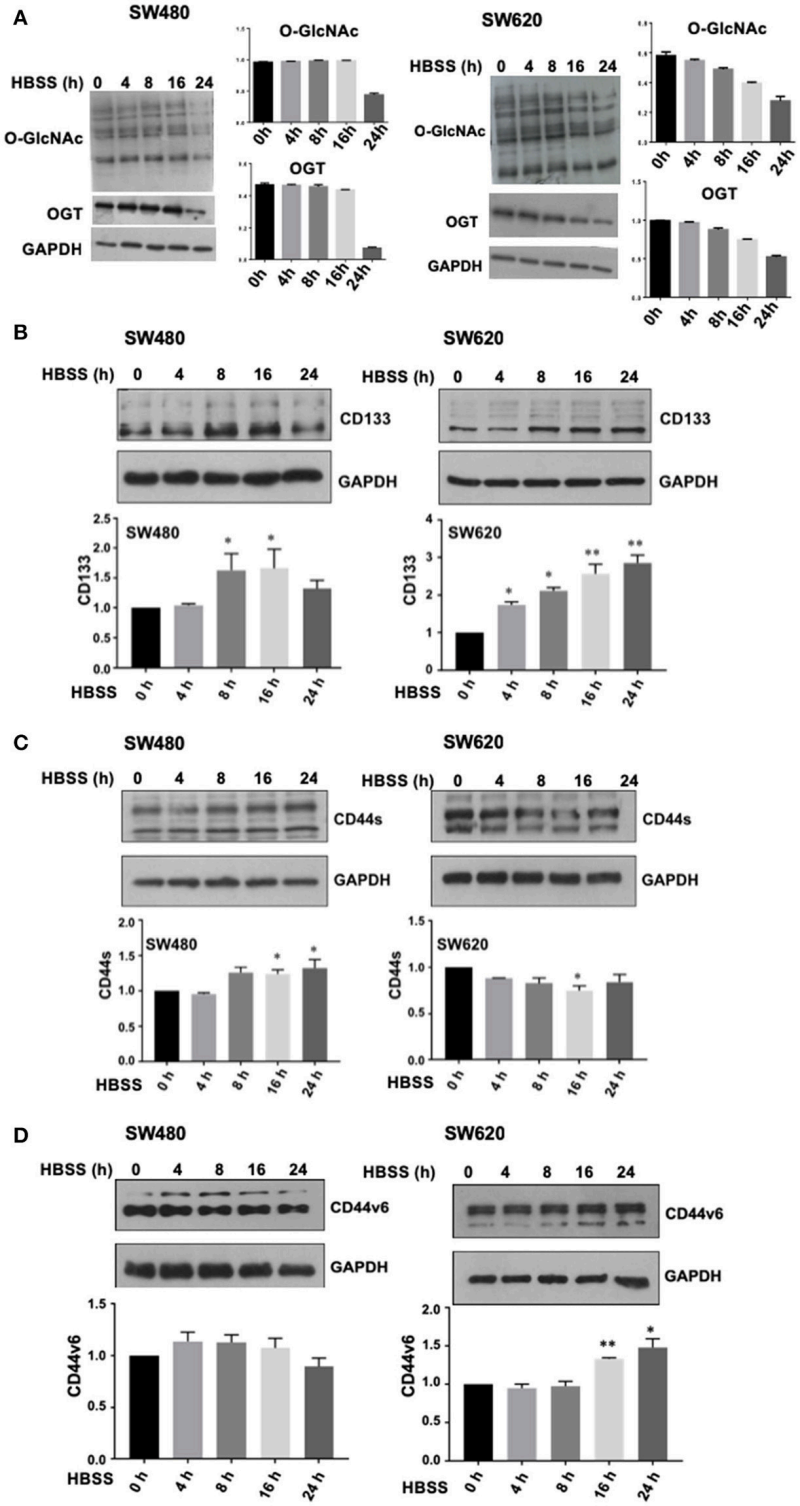
Nutritional stress mimics OGT inhibition effects in cancer stem cells expression. Growth medium from SW480, or SW620 cells, was replaced with Hanks' Balanced Salt Solution (HBSS) for 4, 8, 16, or 24h. Cells were then collected at these time points and assessed for O-GlcNAcylation levels, OGT expression levels, and stem cell markers expression by Western blot. **(A)** O-GlcNAcylation levels and OGT expression levels diminish in a time- dependent manner during incubation of cells in HBSS. Results obtained for CD133 expression are shown in panel **(B)**, CD44 expression in panel **(C)** and CD44v6 expression levels in panel **(D)**. Results shown represent the mean ±SEM of three independent experiments. ^*^p = 0.01; ^**^p = 0.001 (*t*-test) compared with control.

We found that the inhibition of OGT in both primary and metastatic colon cancer cell lines induced not only an increase in stem cell markers expression but also, induced an aggressive phenotype associated with the appearance of double positive stem cell markers subpopulations. Accumulating experimental evidence has shown that microenvironmental stress signals in tumors drive phenotypic plasticity and invasion and determine therapeutic outcome. Nutritional stress, particularly glucose deprivation, would diminish UDP-GlcNAc availability and as a consequence, *O*-GlcNAc intracellular levels. Thus, we hypothesized that exposure of cells to nutritional stress would mimic the effects of OGT inhibition. To this end, growth medium from SW480 or SW620 cells was replaced with Hanks' Balanced Salt Solution (HBSS) for 4, 8, 16, or 24 h (Figure 6). Cells were then collected at these time points and assessed for O-GlcNAcylation levels, OGT expression levels, and stem cell markers expression by Western blot. Results presented in Figure 6A clearly showed that in agreement with our hypothesis, exposure of SW480 or SW620 cells to acute nutritional stress mimicked the inhibition of OGT, since the total O-GlcNAcylation levels and the OGT expression levels were both reduced in a time-dependent manner, being greatly diminished after 16 and 24 h of incubation of cells in HBSS. In addition, results presented in Figure 6B (CD133 expression), 6C (CD44 expression), and 6D (CD44v6 expression), show that indeed, nutritional stress induced a general increase in stem cell marker expression both in SW480 and SW620 cells compared with controls (time 0), that was significant after 8 h of starvation. It is noteworthy that SW480 cells, which under normal culture conditions do not express CD133 stem cell marker, under stressful conditions induce its expression, in addition to increase CD44/CD44v6 expression in a similar manner as when OGT is inhibited. Taken together, these results confirmed that starvation increased the expression of stem cell markers, reinforcing the notion that the activity of OGT is closely integrated with the nutritional status of the cell, and that increased *O*-GlcNAc levels appeared to be part of an endogenous nutrient stress response that is linked to cell survival.

## Discussion

The presence of CSC subpopulations has been identified in nearly all human malignancies. CD133, also called Prominin-1, is a pentaspan transmembrane protein which has been used as a biomarker to identify and isolate stem cells from cancer tissues, including those emerging from colorectal mucosa. The presence of CD133 positive cells have been associated with an aggressive phenotype in several tumor types including CRC. Consistent with this, it has been reported that the CD133+ subpopulation is higher in liver metastasis than in primary colorectal tumors (13). In addition, it has also been demonstrated that CD133+ cells show a high degree of chemoresistance (20, 21). It is interesting to note that in agreement with this, in our study we found that under normal culture conditions, primary SW480 colon cancer cells express the CD44 stem marker and do not express CD133, whereas their derivative metastatic SW620 cell line mainly expresses CD133.

CD44 is a transmembrane glycoprotein which has also been identified as being expressed by many tumor CSCs. It participates in a variety of biological functions such as cell adhesion, tumor cell migration, growth, differentiation, survival, or even in chemoresistance (3, 22, 23). However, CD44s is the smallest and the standard isoform codified by 10 exons, without products of variant additional exons, and the CD44 variants are isoforms expressing additional segments (v2–v10) in the extracellular domain that are generated by alternative splicing (4). Both the standard and the variants can all be recognized by an antibody directed against the standard region but importantly, the expression of CD44 variants has only been found in cancer cells and has been reported as produced during tumor progression (3, 4). Thus, different cells of a tumor can express various, and possibly different sets of CD44 isoforms. In CRC the v6-containing isoform of CD44 is the most frequently found to be associated with metastatic phenotype in the literature (24). It was also found that CD44v6 is involved in acquired drug resistance in CRC (4). According with this notion, while the glandular epithelium of the large bowel expresses the standard form of CD44 but not variant ones, in contrast, highly dysplastic colorectal adenomas, primary and metastatic CRC, express CD44v isoforms (3, 4). In agreement with this, here we found that non-malignant fetal colon 112CoN cells express CD44 but do not express CD44v6, which we found only expressed in colon malignant cells.

In this study we found that in colon cancer cells the inhibition of OGT or the exposure of cells to an acute nutritional stress mimicking the lack of OGT, induce the appearance of an aggressive CD133/CD44 double positive CSC subpopulation. In agreement with our results, these CD133+CD44+ cancer cells have been characterized in several highly metastatic tumors such as CRCs (13–16), HCCs (17), pancreatic cancers (18), and gallbladder carcinoma (19). It has also been reported that in CRC with early liver metastases, co- expression of CD133 and CD44 is significantly higher when compared to those without early liver metastases (15).

To date, the functions played by *O*-GlcNAcylation in stem cells and pluripotency has been poorly investigated and remains unclear. In this respect, Jang et al. (5) have shown that blocking *O*-GlcNAcylation inhibited ESC self-renewal and the efficiency of inducible pluripotent stem cells (iPSC) generation, whereas increasing *O*-GlcNAcylation inhibited normal ESC differentiation. Other authors have also shown that *O*-GlcNAc is required for ESC survival, and that OGT knockout mouse shows embryonic lethality (5, 25). In addition, experimental evidence has revealed that *O*-GlcNAc controls pluripotency by directly regulating transcriptional activities of core components of the pluripotency network. Numerous stem cell factors have been shown *O-*GlcNAcylated such as Oct4 (26) or Sox2 (5). Whereas, the role of Sox2 *O-*GlcNAcylation is still unclear, Oct4 interacts with OGT and is modified in order to regulate pluripotency gene networks (26).

Here we investigated the effects produced by the modification of *O*-GlcNAc levels on the expression of stem cell markers CD44 and CD133 by pharmacological inhibition of OGT or OGA, the enzymes which catalyze the addition and removal of *O*-GlcNAc, respectively. A salient feature obtained here is that we not only confirmed that *O*-GlcNAc serves as a nutrient sensor and the activity of OGT is closely integrated with the nutritional status of the cell, as previously reported in other cell systems, but also that increased *O*-GlcNAc levels appeared to be part of an endogenous stress response that is linked to cell survival. In this respect, accumulating experimental evidence has shown that *O*-GlcNAcylation acts as a nutrient sensor that associates the glucose metabolic status with cellular regulation of signal transduction, transcription, protein function and differentiation (7, 27). As mentioned before, in cancer cells, metabolism is dramatically altered compared with normal cells. They reprogram their metabolism to undergo a high rate of glycolysis and lactic acid fermentation, even under normoxic conditions [Warburg effect (28)]. One consequence of these changes is cellular addiction to glutamine, that in turn, increases the flux through the hexosamine biosynthetic pathway (HBP). Because HBP requires glucose, glutamine, fatty acids, and UTP, is thereby positioned to integrate information on the availability of nutrients (7, 10). Importantly, HBP produces the high-energy-donor UDP- GlcNAc, which is the sugar donor involved in the synthesis of other nucleotide sugars, complex glycosylation and also utilized by OGT to modify target proteins (10, 27).

Increased OGT expression has been detected in numerous cancers, including bladder cancer (29) and lung and colon cancers (30). In addition, HBP enzymes have also been reported to be over- expressed in human prostate cancer patients (10, 31). Here we confirmed that OGT is overexpressed in colon cancer cells compared with non-malignant colon cells. When we decreased *O*-GlcNAc levels as a result of OGT inhibition, we observed, as previously reported, a decrease in cell survival, but unexpectedly, we also observed that the decrease in *O*-GlcNAc levels induced the appearance of an aggressive CD44+/CD133+ small subpopulation which in turn expressed high *O*-GlcNAc levels. In this respect, we found that whereas SW620 double positive stem cells displayed lower levels of *O*-GlcNAc levels than single positive ones as expected, unexpectedly, SW480 double positive stem cell subpopulation displayed higher levels of *O*-GlcNAc compared with their single marker counterparts. But it must be taken into account that the *O*-GlcNAc levels examined in the spheroids derived from the double positive stem cells were obtained from 3-week old 3D spheroids cultures. Thus, a decrease in *O*-GlcNAc levels would be expected to happen only as result of starvation or if glucose were deprived in tumor cells, but once they adapt to the growth conditions, O-GlcNAc levels recuperate as a result of their metabolism. However, it is interesting to note that these levels are lower in the metastatic SW620 cells, compared with the primary SW480 cells from which they derived. Since a decrease in *O*-GlcNAc levels would be expected to happen if glucose were deprived, we reasoned that the exposure of cells to an acute nutritional stress would mimic the effects produced by OGT inhibition. Indeed, our results confirmed that starvation increased the expression of stem cell markers, reinforcing the notion that the HBP pathway and OGT activity are intimately integrated with the nutritional status of the cell and contribute to regulate stemness maintenance.

In this work we also found that the increased *O*-GlcNAc levels observed in colon cancer cells appeared to be part of an endogenous nutrient stress response that is linked to cell survival. In this respect, our data are consistent with the notion that *O*-GlcNAc modification of proteins is a metabolically modulated signaling pathway that regulates cell function and plays a particularly critical role in mediating the response of cells to stress (32). Evidence of this was first reported in 2004, by Zachara et al. when *O*-GlcNAc levels were shown to increase in response to a diverse array of stress stimuli, and inhibition of this response resulted in reduced cell survival (33). Other authors have also shown that *O*-GlcNAc levels are increased in response to stress, that augmentation of *O*-GlcNAc levels conferred increased tolerance to stress (32), and that the acute augmentation of this response is cytoprotective, even in the cardiovascular system (32, 34). Therefore, our data support that *O*-GlcNAcylation modification of proteins not only functions as a nutrient status sensor which plays a critical role in stemness maintenance, but also that it is an important mediator of the response of cells to stressful conditions.

## Ethics Statement

This work has been conducted following the ethical standards according to the Declaration of Helsinki and according to national and international guidelines and has been approved by the Faculty of Medicine Ethical Committee from Universidad Nacional Autónoma de México.

## Author Contributions

MR-F, TL, and GF-G conceived and designed the experiments. GF-G, MC-P, and A-SV-E performed the experiments. MR-F, TL, and GF-G analyzed the data. MR-F and TL contributed reagents, materials, and analysis tools. MR-F wrote the manuscript.

### Conflict of Interest Statement

The authors declare that the research was conducted in the absence of any commercial or financial relationships that could be construed as a potential conflict of interest. The reviewer EZ declared a shared affiliation, with no collaboration, with several of the authors GF-G, MC-P and MR-F to the handling Editor.
